# A Review of Targeted Therapies Evaluated by the Pediatric Preclinical Testing Program for Osteosarcoma

**DOI:** 10.3389/fonc.2013.00132

**Published:** 2013-05-31

**Authors:** Valerie B. Sampson, Richard Gorlick, Davida Kamara, E. Anders Kolb

**Affiliations:** ^1^Nemours Center for Childhood Cancer and Blood Disorders, Alfred I. duPont Hospital for Children, Wilmington, DE, USA; ^2^The Albert Einstein College of Medicine, Bronx, NY, USA

**Keywords:** pediatric preclinical testing program, osteosarcoma, event-free survival, complete response, maintained complete response, partial response, progressive disease with growth delay, progressive disease

## Abstract

Osteosarcoma, the most common malignant bone tumor of childhood, is a high-grade primary bone sarcoma that occurs mostly in adolescence. Standard treatment consists of surgery in combination with multi-agent chemotherapy regimens. The development and approval of imatinib for Philadelphia chromosome-positive acute lymphoblastic leukemia in children and the fully human monoclonal antibody, anti-GD2, as part of an immune therapy for high-risk neuroblastoma patients have established the precedent for use of targeted inhibitors along with standard chemotherapy backbones. However, few targeted agents tested have achieved traditional clinical endpoints for osteosarcoma. Many biological agents demonstrating anti-tumor responses in preclinical and early-phase clinical testing have failed to reach response thresholds to justify randomized trials with large numbers of patients. The development of targeted therapies for pediatric cancer remains a significant challenge. To aid in the prioritization of new agents for clinical testing, the Pediatric Preclinical Testing Program (PPTP) has developed reliable and robust preclinical pediatric cancer models to rapidly screen agents for activity in multiple childhood cancers and establish pharmacological parameters and effective drug concentrations for clinical trials. In this article, we examine a range of standard and novel agents that have been evaluated by the PPTP, and we discuss the preclinical and clinical development of these for the treatment of osteosarcoma. We further demonstrate that committed resources for hypothesis-driven drug discovery and development are needed to yield clinical successes in the search for new therapies for this pediatric disease.

## Introduction

Osteosarcoma (OS), Ewing sarcoma, and rhabdomyosarcoma, are the most common sarcomas in children and adolescents. OS is the most frequent bone cancer occurring mostly in children and adolescents between 10 and 20 years of age (Vander Griend, 1996). It is thought to arise from mesenchymal bone forming tissue and its histological hallmark is the production of malignant osteoid (Mohseny et al., 2009). The metaphyseal growth regions of the long bones of the extremities are the most common site of the primary bone tumor with peak incidences following the adolescent growth spurt and the seventh and eighth decades of life (Marina et al., 2004). Current multimodal therapy consisting of surgery and chemotherapy, achieves a 5-year survival rate of approximately 60–70% in children while patients who present with metastatic disease at diagnosis have a 10–30% survival rate (Ferrari and Palmerini, 2007). The chemotherapy agents which have long demonstrated anti-tumor activity in OS include cisplatin, doxorubicin, ifosfamide, and high dose-methotrexate (Jaffe et al., 1995; Ferrari and Palmerini, 2007). The use of novel effective therapeutic approaches and treatment strategies in patients who are resistant to current therapy could provide an improvement in outcome in patients.

Attempts to identify “druggable” targets in OS tumors have demonstrated a complex karyotype and multiple interacting proteins and pathways implicated in the development of OS. Aneuploidy and inconsistent genetic aberrations have been described in OS (Mohseny et al., 2009), but no single targetable genetic event appears to define this disease. It has been shown that the tumor suppressor p53 is commonly mutated in sporadic OS (Miller et al., 1990). Mutated or deleted p53 alters cellular resistance to DNA damage that can affect the cells capacity to evade the toxic effects of DNA damaging agents. Alterations in genes that regulate p53 including the murine double minute 2 (MDM2), cyclin dependant kinase 4 (CDK4) (Mejia-Guerrero et al., 2010), and the p14 product of the INK4A gene (Benassi et al., 2001) have also been identified in OS. Several groups have demonstrated that mutations in the retinoblastoma (Rb1) gene located on chromosome 13 occurred in a high percentage of OS samples (Wong et al., 1997; Nielsen et al., 1998). High frequencies of allelic loss have been detected at 3q and 18q (Yamaguchi et al., 1992), suggesting that other tumor suppressor genes may be important in OS. In one study conducted by Ebb et al. (2012) overexpression of human epidermal growth factor receptor 2 (HER2/neu, ErbB2) was suggested to be associated with early pulmonary metastases and decreased survival in approximately 40% of cases. Expression of bone morphogenetic protein type II receptor (BMPR2) was also found to correlate with metastasis (Guo et al., 1999). Patients with Rothmund–Thomson Syndrome, associated with a high incidence of OS, have mutations in the RecQ protein-like 4 (*RECQL4*) gene (Simon et al., 2010). In addition, tumors have multiple alterations in gene expression affecting surface receptors, self renewal pathways, angiogenesis, and mechanisms of resistance. OS tumors do not appear “addicted” to specific and identifiable mutations or aberrant pathways, thus presenting a significant challenge in defining potential strategies for the development of targeted therapy.

It is becoming increasingly apparent that few novel targeted agents are capable of achieving single-agent clinical responses despite proof of target inhibition, to justify advancement in clinical development. Understanding the relevant molecular targets, as well as the mechanisms by which sarcomas are able to escape targeted therapies, are critical steps in defining effective clinical applications for new compounds. The establishment of robust preclinical models may be an effective tool to aid in the prioritization of the most effective molecularly targeted therapies. The Pediatric Preclinical Testing Program (PPTP) is an initiative supported by the National Cancer Institute (NCI) made up of a consortium of institutions in the United States and Australia (Houghton et al., 2007). The PPTP has established various models of childhood cancers using panels of xenograft tumors and cell lines for evaluation of *in vivo* and *in vitro* anti-tumor activity of standard and novel agents. Tumor lines include rhabdoid, Wilms tumor and Ewing sarcoma, rhabdomyosarcoma, neuroblastoma, medulloblastoma, ependymoma, glioblastoma, OS, B-cell precursor, and T-acute lymphoblastic leukemia (ALL). Response criteria for the solid tumor panels are categorized as high, intermediate, or low. Agents inducing objective responses [partial response (PR), complete response (CR), or maintained complete response (MCR)] are considered highly active against the tumor xenograft. A PR is defined as ≥50% tumor volume regression, CR is immeasurable tumor volume and MCR is maintained CR at the end of the experimental study (Houghton et al., 2007). Agents inducing stable disease (less than 50% reduction in tumor volume and less than a 25% increase in tumor volume) or progressive disease with tumor growth delay (PD2) are considered to have intermediate activities. Agents producing progressive disease without tumor growth delay (PD1) are considered to have a low level of activity against the tested xenograft (Houghton et al., 2007). These response and activity definitions will be used throughout this review.

Improvements in outcome in pediatric OS have been achieved without the addition of novel agents, but rather through optimization of the dose, combination, schedule, and duration of treatment using standard systemic chemotherapy. Over the last decade, technological advances in research and medicine have provided detailed descriptions of factors that contribute to the malignant phenotype of this disease with the hope of finding new therapeutic treatments and strategies. The recent review of van Maldegem et al. (2012) of published clinical trials for OS shows that most phase III trials are combination treatments of conventional chemotherapy agents. Many biological based treatments evaluated in the PPTP and phase I and II trials have yet to advance to phase III trials. This review summarizes the results of preclinical testing of agents in OS models conducted by the PPTP over the past 6 years (Tables 1 and 2). In particular, we have focused on agents that have demonstrated high and intermediate activities in preclinical OS models and we highlight the outcome of early-phase trials for these targeted therapies. The review discusses trials listed in clinicaltrials.gov and published in PubMed that are informative about the development of novel therapies. Clinical trials were selected if they were specific for pediatric OS or if they enrolled children with OS. Our aim will be to discuss the available clinical data concerning the efficacy and safety of novel agents in pediatric OS, with a focus on those agents evaluated by the PPTP.

**Table 1:** **Agents tested by the PPTP with high (H) and intermediate (I) activities in osteosarcoma xenografts and corresponding clinical trials that include pediatric patients with osteosarcoma**.

Target	Agent	Reference	Response activity	Clinicaltrial.gov identifier
Multiple-tyrosine kinases	Dasatinib	Kolb et al. (2008a)	I	NCT00464620, NCT00788125
VEGFR	Cediranib (AZD 2171)	Maris et al. (2008a)	I	NCT00321581
VEGFR	Sorafenib	Keir et al. (2010)	I	NCT00665990, NCT01518413
VEGFR	Sunitinib	Maris et al. (2008b)	I	–
IGF-1R	SCH 717454	Kolb et al. (2008b)	I	NCT00617890, NCT00960063
IGF-1R	IMC-A12 (Cixutumumab)	Houghton et al. (2010a)	I	NCT00609141, NCT00880282, NCT00831844
IGF-1R	BMS-754807	Kolb et al. (2011)	I	–
mTOR	Rapamycin	Houghton et al. (2008a, 2010b)	H	–
mTOR	AZD8055	Houghton et al. (2012a)	I	–
Aurora kinase A	MLN8237 (Alisertib)	Maris et al. (2010)	H	NCT00739427, NCT01154816
Akt/protein kinase B	MK-2206	Gorlick et al. (2012a)	I	
Akt/protein kinase B	GSK690693	Carol et al. (2010b)	I	–
MEK1/2	AZD6244	Kolb et al. (2010)	I	–
CDK	SCH 727965 (Dinaciclib)	Gorlick et al. (2012b)	I	–
p53	JNJ-26854165 (Serdemetan)	Smith et al. (2012a)	H	–
CENP-E	GSK923295A	Kolb et al. (2008a), Lock et al. (2012)	H	–
HDAC	Vorinostat (SAHA)	Keshelava et al. (2009)	I	NCT00217412, NCT01132911, NCT01422499, NCT01294670
SMAC	LCL161	Houghton et al. (2012b)	I	–
Mitotic spindle	Eribulin	Kolb et al. (2013)	H	
Topoisomerase I	Topotecan	Carol et al. (2010a)	H	NCT01670175
Topoisomerase I	Genz-644282	Houghton et al. (2012c)	H	–

*I, intermediate activity; H, high activity*.

**Table 2:** **Agents tested by the PPTP with low activity (L) in osteosarcoma xenografts and corresponding clinical trials that include pediatric patients with osteosarcoma**.

Target	Agent	Reference	Response activity	Clinicaltrial.gov identifier
Bcl-2/Bcl-XL	ABT-263	Lock et al. (2008)	L	–
EGFR	Lapatinib	Gorlick et al. (2009)	L	NCT01454804
HSP90	AT13387	Kang et al. (2012)	L	–
HSP90	Alvespimycin	Smith et al. (2008)	L	–
Immune system	Lenalidomide	Reynolds et al. (2011)	L	–
MDM2	RG7112	Carol et al. (2013)	L	–
NEDD8-activating enzyme	MLN4924	Smith et al. (2012b)	L	–
PIMI kinase	SGI-1776	Batra et al. (2012)	L	–
Proteosome	Bortezomib	Houghton et al. (2008b)	L	NCT00994500
y-Secretase	RO4929097	Kolb et al. (2012a)	L	–
Unknown	Seneca valley virus	Morton et al. (2010)	L	–

*L, low activity*.

## Agents with Activity in the PPTP Osteosarcoma Panels

### Multi-tyrosine kinase inhibitors

Tyrosine kinases are divided into two main classes, receptor tyrosine kinases (RTKs) and non-RTKs that lack transmembrane domains and are found in the cytosol, nucleus, and inner surface of the plasma membrane (Casaletto and McClatchey, 2012). Tyrosine kinases regulate cellular proliferation, survival, differentiation, function, and motility. Preclinical studies suggest the use of multi-RTKs in OS may be a powerful tool in these tumors which show overexpression of several RTKs including ErbB2, IGF-1R, PDGFR, VEGFR, and activated downstream signal-transduction pathways, as potential targets (Rettew et al., 2012). The prototypical tyrosine kinase inhibitor imatinib, which has dramatically changed the management and improved the prognosis of patients with Philadelphia chromosome-positive chronic myelogenous leukemia (CML) and ALL and has shown efficacy in some solid tumors, including gastrointestinal stromal tumor (GIST) and dermatofibrosarcoma protuberans (DFSP) has demonstrated little evidence of efficacy in OS (Bond et al., 2008). A number of other small-molecule tyrosine kinase inhibitors have been preclinically and clinically evaluated for this disease.

#### Src inhibitor

Src is a non-RTK involved in pathways regulating cell growth, survival, and migration (Creedon and Brunton, 2012). Src is overexpressed in OS and mediates PI3K/Akt anoikis resistance (Diaz-Montero et al., 2006). In OS, inhibition of c-Src prevents cell invasion and induces apoptosis *in vitro* and reduces tumor growth *in vivo* (Akiyama et al., 2008). Dasatinib, a multi-tyrosine kinase small-molecule inhibitor against Src family kinases, which is also approved for first and second line therapy of CML and Philadelphia chromosome-positive ALL (Steinberg, 2007; Aguilera and Tsimberidou, 2009) was tested against the PPTP OS xenograft panels.

These studies by the PPTP demonstrated that dasatinib had intermediate activity in two of six OS xenograft lines (Kolb et al., 2008a) to indicate efficacy in OS. In an *in vivo* model of metastasis, Hingorani et al. (2009) showed effective target inhibition in primary tumors by dasatinib, with no effect on pulmonary metastasis suggesting that the development of pulmonary metastases in OS is independent of Src activation. Lung metastasis is usually associated with poor prognosis in OS patients and these findings along with reports of differences in gene expression patterns between the primary tumor and lung metastasis (Namlos et al., 2012) could aid the development of targeted therapy strategies. Single-agent testing of dasatinib in pediatric patients with OS showed suboptimal responses. In the phase I COG trial, none of the five patients experienced disease stabilization beyond the first course of therapy (Aplenc et al., 2012). The Sarcoma Alliance for Research through Collaboration (SARC) is conducting a phase II study of dasatinib for advanced sarcoma patients 13 years and older (NCT00464620), but this study is not inclusive of patients with OS. Dasatinib is also being investigated in combination with chemotherapy. Ongoing trials include the phase I/II study (NCT00788125) of dasatinib in combination with ifosfamide, carboplatin, and etoposide in young patients with metastatic or recurrent malignant solid tumors, including OS (Table 1). Of note, a SARC randomized trial of another Src inhibitor saracatinib (AZD0530) in patients with recurrent OS localized to the lung is ongoing (NCT00752206).

#### Vascular endothelial growth factor receptor inhibitors

The vascular endothelial growth factor (VEGF) pathway is involved in angiogenesis and is crucial for tumor growth and progression. Several agents for anti-angiogenic therapies have been developed that target either VEGF or its cell surface receptors to induce anti-proliferative, anti-angiogenic, and proapoptotic effects. Of the three VEGF receptor isoforms (VEGFR-1, -2, and -3), VEGFR-3 is overexpressed in OS cell lines and correlates with poor survival (Lugowska et al., 2011). The PPTP has tested several tyrosine kinase inhibitors, three of which show potent and relative selectivity for the VEGF receptor family. These are cediranib (AZD 2171), sorafenib, and sunitinib. Cediranib is an oral bioavailable small-molecule inhibitor. Sorafenib and sunitinib are two broad range kinase inhibitors with similar drug profiles and overlapping targets (PDGFR, FLT3, RET, and KIT) and high selectivity for the VEGFR family. Both drugs are approved by the FDA for treatment of advanced renal cell carcinoma (RCC). Sorafenib is also approved for unresectable hepatocellular carcinoma (HCC) (Keating and Santoro, 2009) and sunitinib is approved for GIST after disease progression or intolerance to imatinib mesylate (Deeks and Keating, 2006).

Cediranib was highly effective in the PPTP OS models with tumor growth delay in four of five xenografts (Maris et al., 2008a). Objective responses (both CR) were achieved in one OS and one rhabdoid xenograft. Combination testing of cediranib with chemotherapy in other xenograft models did not demonstrate additive benefits (Morton et al., 2012), but this approach was not tested for OS. Cediranib was evaluated in combination with the EGFR inhibitor gefitinib in a phase I trial in patients with advanced solid tumors (van Cruijsen et al., 2010) and demonstrated favorable response in one OS patient (PR). In a phase 1 trial and pharmacokinetic study of cediranib in children and adolescents with refractory solid tumors (NCT00321581), objective responses were observed in patients with Ewing sarcoma, synovial sarcoma, and OS (Fox et al., 2010). Notably, bevacizumab, a humanized monoclonal antibody (mAb) targeting the VEGF receptor, has been evaluated in a phase I study in pediatric patients with refractory solid tumors (Glade Bender et al., 2008) and is currently being evaluated in combination with chemotherapy (NCT00458731).

The PPTP testing of sorafenib showed significant tumor growth delay for the OS models (PD2 for five of six tumors) (Keir et al., 2010). A COG phase I study of sorafenib (Widemann et al., 2012) was successfully completed and a phase II trial is in progress. In a phase II trial conducted by the Italian sarcoma group in a cohort of 35 patients greater than 14 years with OS (Grignani et al., 2012), 14% of patients had objective responses (3PR, 2MR) and 29% of patients had SD. Sorafenib is also being clinically investigated in combination with bevacizumab (NCT00665990) and irinotecan (NCT01518413).

Sunitinib demonstrated effective tumor growth inhibition comparable to sorafenib in the PPTP OS models (PD2 for three of six tumors) (Maris et al., 2008b). However, the evaluation of sunitinib in the COG phase I and pharmacokinetic study in pediatric patients with refractory solid tumors did not achieve objective responses. Stable disease was reported for one OS patient in this study (Dubois et al., 2011, 2012), but concerns for cardiac toxicity have limited interest in additional trials. Further preclinical testing of these VEGF-targeted agents in combination with conventional chemotherapeutic regimens may enhance the efficacy of these drugs in the treatment of OS tumors.

#### Insulin-like growth factor-1 receptor inhibitors

The insulin-like growth factor-1 receptor (IGF-1R) is a transmembrane RTK for which selective IGF-1R tyrosine kinase inhibitors have been developed. The binding of insulin-like growth factor-1 and 2 (IGF-1 and IGF-2) to the IGF-1R results in autophosphorylation and subsequent activation of multiple signaling pathways including the Ras/Raf/mitogen-activated protein kinase (MAPK) and PI3K/Akt pathways which both regulate cell growth and development (Tognon and Sorensen, 2012). IGF-1R has been implicated in the development of sarcomas and inhibition of IGF-1R function has been demonstrated to reduce growth in OS, rhabdomyosarcoma, and Ewing sarcoma cell lines (Toretsky et al., 1997; Scotlandi, 2006). More than 25 antibodies and small molecules that specifically inhibit IGF-1R have undergone preclinical and clinical testing in both adults and children. Of these, two fully humanized mAbs SCH 717454 and cixutumumab (IMC-A12) and one small-molecule ATP-competitive inhibitor of IGF-1R, BMS-754807 have been evaluated by the PPTP.

*In vivo* activity of SCH 717454 was demonstrated by tumor regression (MCR) in two lines and tumor growth inhibition (PD2) in another two of six OS xenografts (Kolb et al., 2008b). Wang et al. (2010) also showed similar tumor regression by SCH 717454 in an OS model, with enhanced anti-tumor effects when used in combination with cyclophosphamide and cisplatin. Preliminary evidence of anti-tumor activity was reported in a phase II study of SCH 717454 in subjects with relapsed OS or Ewing sarcoma after standard therapy (NCT00617890). A planned trial of SCH 717454 in combination with chemotherapy in patients with solid tumors was recently terminated (NCT00960063).

When evaluated by the PPTP, the mAb cixutumumab demonstrated single-agent activity *in vivo* in OS, and induced intermediate responses (PD2) in three of five OS xenografts (Houghton et al., 2010a). Combination (stage 2) testing of cixutumumab with rapamycin resulted in superior responses compared to the single agents alone (Kolb et al., 2012b). In a phase II COG trial cixutumumab was also well-tolerated as single-agent therapy in patients with solid tumors (Malempati et al., 2012). Limited single-agent activity of cixutumumab was seen mainly in Ewing sarcoma with a response rate of approximately 10%. The recent study in combination with mammalian target of rapamycin (mTOR) inhibitor temsirolimus in the relapsed/refractory setting (NCT00880282) also demonstrated best single-agent activity in Ewing sarcoma tumors (Naing et al., 2012). In the phase II trial which included patients with OS, the combination of cixutumumab and temsirolimus showed clinical activity in patients, but did not have an effect on median progression-free survival (PFS) (Schwartz et al., 2013). However, despite clear evidence of clinical benefits in some pediatric patients, several drug companies have curtailed or eliminated anti-IGF-1R programs because the observed clinical benefits of targeting this pathway in single and multi-agent strategies did not met the primary endpoints in many adult trials. The current development of anti-IGF-1R therapy is being reviewed.

BMS-754807 showed good intermediate response against the PPTP OS cell lines and induced significant differences in event-free survival (EFS) in xenografts (PD2 in four of six tumors) (Kolb et al., 2011). Currently, this compound is not in clinical development for pediatric malignancies. These findings highlight current challenges for future opportunities in anti-IGF-1R developmental therapies and warrant the investigation of combination and dosing strategies along with molecular studies of mechanisms of response to achieve broader activity and greater toxicities with these therapies.

### mTOR inhibitors

mTOR is a serine/threonine kinase intermediary within the PI3K/Akt pathway that regulates protein synthesis, cell proliferation, survival, and angiogenesis. The mTOR complex consists of mTOR complex-1 (mTORC1) which regulates cellular proliferation and mTOR complex-2 (mTORC2) which phosphorylates and activates Akt. Activation of the mTOR pathway is implicated in the development and progression of OS (Wan et al., 2005) which makes mTOR an appealing target for therapeutic intervention. Rapamycin (sirolimus) and rapalogs (everolimus, temsirolimus, and ridaforolimus) are immunosuppressive macrocyclic lactone antibiotics that inhibit the kinase activity of mTORC1 while mTORC2 is generally rapamycin-insensitive (Yung, 2010).

Response activity to rapamycin was high in one OS xenograft (CR) and intermediate in five tumors (PD2) (Houghton et al., 2008a). Furthermore, the addition of rapamycin to either cyclophosphamide or vincristine resulted in enhanced responses in the OS models (Houghton et al., 2010b). A phase II trial for treatment of advanced sarcoma patients with rapamycin and cyclophosphamide, did not meet the primary endpoint of greater than 25% 6-month PFS rate (Schuetze et al., 2012). Initial reports for everolimus, temsirolimus, and ridaforolimus (Spunt et al., 2011) have provided evidence of favorable anti-tumor activity in patients with a broad range of solid tumors. The randomized phase II trial of ridaforolimus as single-agent therapy met the primary endpoint of improved PFS in two OS patients (Chawla et al., 2012). More recently in a phase III sarcoma maintenance study (Blay et al., 2011), ridaforolimus met the study endpoint of a statistically significant improvement in PFS, compared with a placebo group (hazard ratio = 0.72, *P* = 0.0001, stratified log-rank) though specific results in OS are not yet available. Ridaforolimus (an analog of the drug sirolimus) is under clinical development for numerous adult cancers but is not FDA approved. A phase II trial of pazopanib and everolimus in patients with PI3K mutations and/or phosphatase and tensin homolog (PTEN) tumor suppressor loss is underway (NCT01430572).

It is likely that in response to inhibition to mTORC1, mTORC2 may compensate for loss of mTORC1 activity through activation of Akt, leading to acquired resistance to rapamycin and rapalogs (Gulhati et al., 2011). The dual mTOR1/mTOR2 inhibitor AZD8055 demonstrated low activity *in vivo* against the PPTP OS xenografts as PD1 was reported for five of six tumors (Houghton et al., 2012a). AZD8055 is not currently in clinical development while other novel small-molecule inhibitors that are mTOR-selective or dual mTOR/PI3K inhibitors have entered clinical trials. A key focus in the development of these drugs will be to establish the effectiveness of combination strategies with other targeted therapies and standard cytotoxic agents. IGF-1R signals through Akt and is suggested to be involved in resistance to mTOR inhibitors. Combined inhibition of IGF-1R and mTOR has been evaluated. Antibodies targeting IGF-1R (R1507, figitumumab, cixutumumab) in combination with rapamycin and rapalogs showed enhanced activity compared to the single agents (Juergens et al., 2011; Quek et al., 2011; Kolb et al., 2012b). The clinical testing of temsirolimus with cixutumumab in 27 patients demonstrated prolonged CR in one patient with Ewing sarcoma and PR in four patients (Naing et al., 2012). The phase I study of everolimus and CP-751871, a fully human anti-IGF-1R mAb, achieved SD in patients with sarcoma and other solid tumors (Quek et al., 2011). Further use of mTOR inhibitors in the treatment of OS patients is under investigation as single-dose therapy [everolimus (NCT01216826), sirolimus (NCT01331135)] and multi-agent therapy [sirolimus and cyclophosphamide (NCT00743509)] with the major goals of determining effective dosing schedules and additive combinations.

### Aurora a kinase inhibitor

The ability of cancer cells to evade death is one of the hallmarks of cancer development and loss of cell cycle control leads to deregulated cell proliferation in many tumors including OS. Aurora kinases regulate cell cycle transit from G2 through cytokinesis. Three mammalian aurora kinase genes encode aurora A, B, and C proteins and inhibition of any of these potentially results in abnormal mitotic events and apoptosis. Overexpression of aurora kinases has been correlated with poor prognosis in canine OS, which his high similarities to the human disease (Mueller et al., 2007). Currently there are about 30 aurora kinase inhibitors in different stages of preclinical and clinical development (Kollareddy et al., 2012). MLN8237 (alisertib) was tested by the PPTP (Maris et al., 2010). Evidence of intermediate to high anti-tumor activity was demonstrated, as MCR was achieved in one of six OS tumor xenografts *in vivo* and intermediate activity was observed for the other five. This effect was also seen in neuroblastoma tumors (three of six). A current COG pediatric phase 1/II study investigating MLN8237 in children with relapsed/refractory solid tumors (NCT00739427) includes a cohort of patients with OS. Dose limiting toxicities of aurora kinase inhibition for neuroblastoma has been demonstrated (Shang et al., 2009). Of note, in one study, canine OS cells exhibit resistance to aurora kinase inhibitors (Cannon et al., 2013). Better biological understanding of tumor specific variations in drug sensitivity to aurora kinase A inhibitors may also guide important preclinical evaluation of single and multi-agent therapies in OS.

### Akt/PKB inhibitors

PI3K and Akt activation may drive tumorigenesis in OS by regulating effectors that are different from mTORC1. Inhibition of Akt signaling is anticipated to reduce tumor survival and enhance the effectiveness of cytotoxic chemotherapy by increasing apoptosis (Diaz-Montero et al., 2006; Carol et al., 2010b). The development of specific inhibitors of Akt is challenging since Akt belongs to the AGC family of kinases and shares homology with PKA and PKC. Furthermore, human Akt has three isozymes that show >85% homology (Akt1, Akt2, and Akt3) but distinct functionality. Specificity as well as isozyme selectivity of Akt inhibitors will be critical for optimal anti-apoptotic effects of tumor cells *in vivo*. MK-2206, a highly selective non-ATP-competitive allosteric Akt inhibitor and GSK690693 a novel ATP-competitive, pan-Akt kinase inhibitor selective for the Akt isoforms were two agents tested for anti-tumor activity by the PPTP (Carol et al., 2010b; Gorlick et al., 2012a).

MK-2206 showed significant differences in EFS distribution *in vivo* against OS xenografts including PD2 in two tumors, but no objective responses (Gorlick et al., 2012a). Of note, triciribine, a nucleoside analog that causes dephosphorylation of active Akt was evaluated for adult OS but showed no responses and unexpected toxicity (Garrett et al., 2011). One explanation is that activation of Ras/Raf signaling pathways and the mTOR signaling cascade, may limit the efficacy of Akt/PKB targeted therapy (Carol et al., 2010b), and suggests that the feedback phosphorylation of Akt and activation of the PI3K/Akt pathway may be a mechanism for resistance. Concomitant disruption of these mechanisms may be an effective strategy for the development of novel anti-cancer therapies. These findings reiterate the need for extensive preclinical testing of these agents and the development of predictive biomarkers of response. The Akt inhibitor, GSK690693 was reported to have modest anti-tumor activity in the PPTP testing as intermediate activity against two of six OS xenografts was demonstrated (Carol et al., 2010b). One planned study to investigate GSK690693 in pediatric subjects with relapsed or refractory hematologic malignancies was withdrawn prior to patient enrollment and this agent has not been clinically studied for any pediatric malignancies.

### MEK1/2 inhibitor

MAPK or extracellular receptor kinase (ERK) is activated by MAPK/ERK kinase (MEK). MEK1 and MEK2 are dual-specificity protein kinases that function in the MAPK signaling cascade to control cell growth and differentiation (Bromberg-White et al., 2012). MEK1/2 is stimulated by a wide variety of growth factors and cytokines and also by membrane depolarization and calcium influx. Ras/Raf is upstream of MEK and aberrant activation of the Ras/Raf/MEK/MAP kinase pathway (MAPK pathway) is associated with the pathogenesis of many cancers including OS lung metastasis (Yu et al., 2011). Many growth factor receptors associated with the biology of OS (IGF-1R, EGFR, VEGFR, and PDGFR) activate the MAPK/ERK pathway to make it a powerful target for therapeutic intervention. AZD6244 is a potent and specific inhibitor for MEK 1/2 and demonstrated effective tumor growth inhibition in the PPTP OS panel. AZD6244 demonstrated intermediate activity in three of six OS xenograft lines (Kolb et al., 2010). This compound is also in development for pediatric glioma through the Pediatric Brain Tumor Consortium (PBTC) and is currently in phase II testing of pediatric patients with melanoma but has not yet entered the pipeline for clinical development for pediatric sarcomas. There are other MEK inhibitors including trametinib (GlaxoSmithKline) and pimasertib (MERK) in clinical development for patients with melanoma.

### Cyclin-dependent kinase inhibitor

Cyclin-dependent kinases regulate orderly progression through the cell cycle. Activated by association with cyclins, Cdks phosphorylate key regulatory substrates such as the Rb protein (Stone et al., 2012). CDKs are frequently upregulated in human cancers along with overexpression of cyclin partners or inactivation of CDK inhibitors. Inhibitors of CDKs such as seliciclib and alvocidib induce the apoptosis of several tumor cells. Dinaciclib (SCH 727965) is capable of specific inhibition of CDKs with low nanomolar potency against CDK1, CDK2, CDK5, and CDK9 and induces cell cycle arrest and apoptosis (Parry et al., 2010). Interest in CDKs as a therapeutic target stems from the central role in cell cycle regulation, the modest toxicity of this class of chemotherapy, activity against CLL and the existing preclinical data in adult histotypes (Gorlick et al., 2012b). When tested against the PPTP OS panels, stable disease was observed in one xenograft. A separate study has shown that dinaciclib-induced apoptosis of several OS cell lines was p53-independent (Fu et al., 2011). Dinaciclib is not currently in clinical development for pediatric malignancies but these findings support further preclinical testing of this novel small-molecule multi-CDK inhibitor in combination therapies to enhance the anti-tumor activity in OS cells.

### p53 inhibitor

The transcription factor and tumor suppressor p53 is activated in cells in response to DNA damage. MDM2 (mouse double minute 2) binds and inactivates p53 to promote ubiquitination and proteasomal degradation for the p53 growth-suppressive function in unstressed cells. Selective inhibitors have been developed which disrupt binding of p53 and MDM2, to activate the p53 pathway in cancer cells leading to cell cycle arrest and apoptosis. Mutations in p53 (Miller et al., 1990) and amplification of MDM2 have been reported in OS, though with relatively low incidence in comparison to adult malignancies (Mejia-Guerrero et al., 2010). Serdemetan (JNJ-26854165) was originally developed as an MDM2 antagonist that activates the p53 protein to induce apoptosis in cancer cell lines. In the PPTP testing, this agent demonstrated tumor growth inhibition in the OS models (Smith et al., 2012a). An objective response was observed in one xenograft in the OS panel (MCR) and intermediate activity (PD2) was observed in another tumor. In a separate study, OS cells that express wild type p53 were sensitive to nutlin-3 which also targets the p53-MDM2 axis (Wang et al., 2012). A phase I clinical trial for serdemetan in adults with advanced solid tumors reported no objective responses and prolonged SD in three patients (Tabernero et al., 2011). This agent has not been studied in pediatric OS, but future testing of MDM2 antagonists for the treatment of this disease can be explored. Additionally, since Akt also activates MDM2 and inhibits p53, combination strategies with Akt inhibitors may have additive benefits in OS.

### Centromere-associated protein E inhibitor

The centromere kinesin motor protein (CENP-E) is a recent candidate for molecular targeting in cancer. CENP-E is required for correct chromosomal alignment in mitosis. This protein integrates mitotic spindle mechanics with mitotic checkpoint signaling and is overexpressed in a variety of human tumors suggesting a role in tumor cell proliferation. Inhibition of CENP-E in cultured human tumor cells leads to cell cycle arrest in mitosis with bipolar mitotic spindles and misaligned chromosomes and eventual cell death. In the PPTP testing of GSK923295A, two OS xenografts met the criteria for high activity with a CR or MCR (Lock et al., 2012). These results demonstrate that CENP-E may be a valuable target in OS and suggest further *in vivo* evaluation in the context of tolerable doses achievable in humans. GSK923295A is currently in a phase I clinical trial for adults with solid tumors however this agent is not in clinical development for pediatric OS patients.

### Histone deacetylase inhibitor

Histone deacetylases (HDACs) regulate acetylation of histone lysine residues which triggers transcriptional activity promoting cell transformation and proliferation. Deregulation of histone acetylation is associated with disease pathogenesis by promoting alterations in chromatin structure and gene transcription. HDAC inhibitors induce growth arrest and cell death by epigenetic changes in many preclinical and clinical cancer models. Vorinostat is an oral HDAC inhibitor of class I and II HDACs that is in clinical trials for hematologic and solid tumors that metastasize and compromise bone structure. Vorinostat is approved for cutaneous T-cell lymphoma and has advanced to phase III clinical trials for multiple myeloma. It is also in many phase II trials including acute myeloid leukemia, B-cell lymphoma, glioblastoma, myelodysplastic syndromes, and breast cancer.

Preclinical studies of vorinostat in the PPTP revealed intermediate activity (PD2) in two of four OS tumors (Keshelava et al., 2009). Vorinostat has been tested in combination with 13-cis retinoic acid (13cRA) in a COG study in children with refractory solid tumors (Fouladi et al., 2010) and in preclinical medulloblastoma models (Spiller et al., 2008). A phase I clinical trial testing vorinostat with and without isotretinoin (NCT00217412), and another COG trial testing vorinostat in combination with bortezomib in children with refractory or recurrent solid tumors (NCT01132911) (Muscal et al., 2013) were recently concluded. In these studies, only a moderate effect of vorinostat was observed and treatment of metastatic tumors with vorinostat had limited success. Current phase II studies enrolling pediatric OS patients include the testing of vorinostat in children (NCT01422499) and in combination with etoposide in pediatric patients younger than 21 years at diagnosis with refractory solid tumors (NCT01294670). A role for HDACs in deregulating Notch pathway expression was shown to contribute to the ability of OS cells to metastasize in one study. A better understanding of the effects of HDAC inhibitors in combination with epigenetic and DNA damaging drugs may be essential in planning therapies with vorinostat and other signal-transduction pathway inhibitors in OS.

### Second mitochondria-derived activator of caspases

The elucidation of the structure and function of the second mitochondria-derived activator of caspases (Smac) protein has triggered the development of Smac derivatives and mimetics for use in cancer treatment. During programed cell death, Smac, an endogenous proapoptotic protein of the mitochondria is released and promotes apoptosis via inhibition or proteasomal degradation of the inhibitor of apoptosis (IAP) family of proteins. Included in the IAP family is X-linked IAP protein (XIAP) and cellular-linked IAP protein-1 and -2 (cIAP1 and cIAP2) (Chen and Huerta, 2009). Smac mimetics induce the auto-ubiquitylation and proteasomal degradation of cIAP1 and cIAP2 to increase nuclear factor κB (NF-κB) signaling and tumor necrosis factor-α (TNFα) production leading to caspase-8 activation and apoptosis. Overexpression of Smac has been shown to sensitize OS cells *in vitro* to chemotherapeutic drug-induced apoptosis (Hotta et al., 2003). Thus, the use of small-molecule drugs that act as Smac mimetics in OS cells that overexpress IAPs or underexpress Smac might be a potent strategy to elicit a proapoptotic anti-cancer response.

LCL161 is a small-molecule Smac mimetic which induces apoptosis in some cancer cell lines and potentiates the effects of tyrosine kinase inhibition (Houghton et al., 2012b). LCL161 demonstrated tumor growth delay in five of six OS xenografts and intermediate activity in one xenograft in the PPTP. A phase I, multi-center, open-label, dose-escalation study of oral LCL161 in adult patients with advanced solid tumors was recently completed (NCT01098838). A phase I study of safety and efficacy in combination with paclitaxel in with advanced solid tumors is ongoing. LCL161 has not been studied in pediatric patients with OS. The future application of small-molecule Smac mimetics as a treatment for OS warrants further investigation in multi-agent therapies with chemotherapy or other targeted therapies inclusive of agents that target apoptosis.

### Targeting DNA and mitosis

In a tumor marked by aneuploidy, targeting the mitotic spindle and DNA repair are considered to be reasonable therapeutic approaches. While typically considered non-targeted or conventional chemotherapies, mitotic spindle poisons and topoisomerase inhibitors are included in this review. Vincristine was highly active against one OS xenograft (Houghton et al., 2007), while eribulin, a novel, fully synthetic compound capable of irreversible mitotic blockade showed high and intermediate activity against five of six OS xenografts in the PPTP (Kolb et al., 2013). Eribulin is currently approved for the treatment of recurrent metastatic breast cancer.

Topoisomerases maintain the helical structure of DNA. Two topoisomerase inhibitors topotecan a small-molecule DNA topoisomerase I poison (Anderson et al., 2008) and Genz-644282, a novel non-camptothecin topoisomerase I inhibitor that is already in clinical development for adult malignancies, were tested by the PPTP. Topotecan achieved intermediate activities (PD2) in all six OS xenografts (Carol et al., 2010a). A phase I study by COG evaluated topotecan administered by 21-day continuous infusion of topotecan in children with relapsed or refractory solid tumors. Of the 11 OS patients enrolled, one demonstrated SD and nine had PD (Hawkins et al., 2006). The development of topotecan in multi-agent therapy is suggested. Following treatment with the Genz-644282 inhibitor, four of five OS xenografts showed objective responses and the fifth showed PD2 (Houghton et al., 2012c) in PPTP testing. A phase I dose-escalation study to assess the safety and tolerability of Genz-644282 in adult patients with solid tumors is in progress but pediatric patients are not enlisted.

## The Progressive Search for Targeted Therapies in Osteosarcoma

The identification of new therapeutic drugs using strategies which target cell proliferation (multi-tyrosine kinase receptor inhibitors), angiogenesis (VEGF inhibitors), apoptosis, and DNA cytotoxicity in combination with standard chemotherapy has demonstrated promise in preclinical and early-phase clinical trials for OS. Significantly, these studies also highlight many of the major challenges faced by clinicians and researchers in the development of novel drugs and therapies for this disease. Many signal-transduction pathways activated by binding of growth factors to their receptors are proven “druggable” targets. However, the responses are variable and the most dramatic benefits seen with biologic agents in ongoing studies are in the adjuvant setting. It is now apparent that successful treatment for this heterogeneous disease will involve combination strategies of new agents or personalized therapies. This integrated approach is anticipated to help realize the true potential of targeted therapies in OS.

The emergence of numerous anti-cancer agents and the small number of OS patients eligible for early-phase clinical trials present another challenge in the clinical testing of novel compounds. Predictive animal model systems for preclinical studies of safety assessment, drug metabolism, and pharmacokinetics may help to inform and prioritize available drugs for clinical development. The systematic preclinical evaluation of new agents in pediatric cancer has received tremendous support by the efforts of the NCI-supported PPTP (Houghton et al., 2007). Since 2007, this program has facilitated the comprehensive analysis of more than 50 novel and standard agents in *in vitro* and *in vivo* panels of childhood cancers.

The PPTP has demonstrated that OS xenografts provide a rapid and efficient platform for the testing of new therapies for anti-tumor effects. Given the complex genomic changes that are associated with this disease, validation of xenografts is necessary to ensure the use of robust OS model systems and to reduce discrepancies and errors in molecular studies and drug screening. Gene expression profiling of the *in vitro* and *in vivo* OS panels of the PPTP has shown that gene expression patterns are preserved in OS cell lines and xenografts (Houghton et al., 2007). Consequently, the activity of an agent in these preclinical models of OS is considered controllable, reproducible, and potentially informative of clinical activity in patients. However, there are notable exceptions to these expectations. The PD results of the phase II study of continuous-infusion topotecan (Hawkins et al., 2006) did not reflect the favorable response reported in the PPTP testing (intermediate activity) (Carol et al., 2010a). Different dosing schedules and pharmacokinetics in mice and humans and the interpretation of data may result in false positive and false negative xenograft results. Nonetheless, OS xenograft models offer a link between preclinical and clinical testing with the potential of benefits to pediatric patients.

The PPTP is testing the hypothesis that OS xenografts can predict activity in clinical testing for some OS patients (Table 1). Phase I and II clinical trial results for cixutumumab, sorafenib, and rapamycin have demonstrated that these preclinical modeling systems are effective (Chawla et al., 2012; Grignani et al., 2012; Malempati et al., 2012) as these agents with high levels of response in the PPTP are well-tolerated and demonstrated promising anti-tumor activity in some adult and pediatric patients. Importantly also, the PPTP has tested several agents with high clinical successes in the treatment of other malignancies which do not yield similar responses in OS (summarized in Table 2). These include the inhibitor lapatinib which has clinical efficacy against ErbB2-overexpressing breast cancer but showed little single-agent activity against the xenografts of the PPTP *in vivo* panel (Gorlick et al., 2009). Further, in contrast to serdemetan, the p53-MDM2 inhibitor RG7112 (Carol et al., 2013) did not show significant tumor regression in the PPTP OS tumors highlighting the differences in potencies and toxicities of similarly targeted drugs (Table 2). Insufficient proof of target inhibition in preclinical testing may also suggest limited clinical benefits of such agents. Establishing these critical elements of efficacy and toxicity is expected to avert the clinical testing of ineffective agents and assist in the prioritization of agents for OS drug discovery and development.

The screening of multiple patient-derived OS xenografts generated from different cell lines in the PPTP panels also leverages the molecular diversity in OS to provide comparative screening for improved drug evaluation. The MCR and PD2 responses in the *in vivo* OS xenografts following anti-IGF-1R targeted therapy (Kolb et al., 2008b) demonstrate clear heterogenous responses in these tumors. These preclinical responses are significant since variable responses were also observed in early-phase clinical trials using the fully humanized mAbs SCH 717454, cixutumumab, and R1507 (Wang et al., 2010; Pappo et al., 2011; Malempati et al., 2012). The PPTP panels therefore provide a framework for evaluating models of mechanisms of action and mechanisms of resistance for individual agents. Further preclinical experiments which focus on activation of oncogenic signaling pathways that maintain aberrant cell proliferation can be studied in these tumors. Evidence of acquired resistance through activation of the PI3K/Akt pathway has been described in the evaluation of sorafenib, IGF-1R, and mTOR inhibitors as monotherapy in the preclinical setting (Juergens et al., 2011; Quek et al., 2011; Pignochino et al., 2013). Other resistance mechanisms may not be specific to an inhibitor, but rather reflect a compensatory response to toxic stimuli. Preclinical studies of mechanism and response would aid in framing decisions when translating these targeted therapies to the clinic.

By design, some preclinical testing strategies present inherent limitations. Almost all mAbs in clinical trials are either chimeric or humanized antibodies produced in transgenic mice. Thus the fully human monoclonal anti-GD2 antibody (ch14.18) which targets the ganglioside GD2, that is expressed on the surface of OS and Ewing sarcoma cells and denosumab which binds and neutralizes the activity of the receptor activator of nuclear factor kappa B ligand (RANK-L) cannot be tested in mice. Canine models recapitulate the primary human disease and provide a useful platform for the development of more effective treatments for both humans and canines. Spontaneous OS is the most common primary bone tumor in dogs with many phenotypic and molecular similarities between dogs and humans (Mueller et al., 2007). Of note, the majority of *in vivo* OS models have addressed the conventional, high-grade types of OS which are the most frequently occurring subtypes of this disease. Rarer subtypes which include low-grade, small cell, telangiectatic, periosteal, and high-grade surface OS which present with distinguishing genetic features may have different drug responses to conventional tumors (Martin et al., 2012). The use of preclinical models for the identification of accurate biomarkers of response and resistance that correlate changes of the molecular target to the drug treatment will serve as key elements in the successful translation of preclinical results to the clinical setting.

In summary, the goal to find new targeted therapies for patients with OS has seen the development of suitable *in vivo* models and advanced testing strategies for the evaluation of different targets and therapies. A wealth of preclinical and clinical data of agents with activity in OS now exists which provides support for novel, single-agent therapies currently in clinical development. Future work evaluating these agents in the preclinical models can focus on their utility in novel, multi-agent combinations, to justify future approaches that may result in improved prognosis of pediatric patients. The search for new treatment strategies is ongoing and there remains considerable hope that progressive and continuous research through these preclinical testing programs will fully exploit the potential of targeted therapies in OS to maximize the benefits in this pediatric disease.

## Conflict of Interest Statement

The authors declare that the research was conducted in the absence of any commercial or financial relationships that could be construed as a potential conflict of interest.
